# Improvement of Alternaria Leaf Blotch and Fruit Spot of Apple Control through the Management of Primary Inoculum

**DOI:** 10.3390/microorganisms11010101

**Published:** 2022-12-30

**Authors:** Jordi Cabrefiga, Maria Victoria Salomon, Pere Vilardell

**Affiliations:** Sustainable Plant Protection, Intitute of Agrifood Research and Technology, La Tallada d’Empordà, 17134 Girona, Spain

**Keywords:** Alternaria leaf blotch, Alternaria fruit spot, *Trichoderma*, spore release, inoculum management

## Abstract

*Alternaria* spp. is the causal agent of apple leaf blotch and fruit spot, diseases of recent appearance in Spain. The overwinter inoculum of *Alternaria* spp. is the source of primary infections in apple, thus the aim of this work was to optimize the control of infection through two environmentally friendly inoculum-management strategies, the removal of winter fallen leaves and the treatment of leaves with the biological agent *Trichoderma asperellum* to inhibit or prevent inoculum development in commercial orchards. The results of commercial orchard trials showed that leaf aspiration and application of *T. asperellum* on the ground have efficacy to reduce fruit spot between 50 and 80% and leaf blotch of between 30 and 40% depending on the year. The efficacies on the reduction of leaf blotch were slightly lower than of fruit spot. Disease reduction has been related to a reduction of total spores released during the season. Results of dynamics of spore release indicate that factors influencing spore release were rainfall and temperature. In conclusion, the use of environmentally friendly strategies combined with standard fungicides, and with monitoring environmental conditions, might allow a reduction in the number of phytosanitary applications, thus achieving the goal of reducing their use.

## 1. Introduction

*Alternaria* is a widespread genus of fungi that includes several species of saprophytes and pathogens of different plant species including a wide variety of crops during pre- and postharvest, thus causing production losses that have a significant economic impact on agriculture. In apples (*Malus domestica* Borkh.), some species of *Alternaria* have been reported as responsible for damage in leaves as well as in young and mature fruits causing leaf blotch, fruit spot, and moldy core diseases in susceptible varieties such as Fuji, Royal Gala, Red Delicious and Golden Delicious [1,2,3]. The first pathology was associated with the species *Alternaria mali* (Roberts) (syn. *Alternaria alternata* f. sp. *mali*, or *Alternaria alternata* apple pathotype) [4] and was first identified in 1924 in the USA [5]. Although multiple *Alternaria* species have been described as causing leaf blotch and fruit spot of apple, *A. alternata* and the *A. arborescens* species complexes have been commonly associated with them [3,6,7,8]. These studies indicate that the distribution of *Alternaria* species causing leaf blotch and fruit spot in apple is different among regions, though *A. mali* is still the most commonly cited pathogen worldwide [9]. In Spain, three species have been found to be associated with these diseases, including *A. arborescens*, *A. tenuissima* and *A. alternata* (this work, data not shown), as it has been described in Australia [10], in Italy [8], in Israel [3] and more recently in France [11].

The occurrence of Alternaria leaf blotch and fruit spot has been reported in all apple-producing regions of the world [11], being one of the most important diseases of apple in Southeast Asia, including Japan, South Korea and China [9], southeastern USA [9] and Australia [10]. In Europe, Alternaria leaf blotch and fruit spot were first reported in 1996 in Yugoslavia [12] and later described in some important apple growing areas such as France, where they started to be a relevant problem in 2016 [11], Netherlands where they were detected in a survey in 2014 [6], and Italy, where the problem dates back to 1999 [13]. In Spain, the first symptoms, affecting mainly Gala and Golden Delicious varieties, were observed in the northeastern part in 2006, although damages of concern appeared in 2014–2015 [14]. In this region, apple is an important fruit crop with a total production area of 2720 ha and a production of 101,745 T in 2021 [15]. Since the first focus, Alternaria leaf blotch and fruit spot have spread throughout the area to affect more than 20% of commercial orchards of sensitive varieties, including Golden Delicious, Gala and Pink Lady, especially.

The first symptoms appear on leaves at the end of May as small circular purplish-brown spots of 3 to 5 mm diameter bordered by a dark margin that evolve into irregular darker lesions. Spots increase in number and size during the season. When severe infection occurs in petioles, the leaves turn yellow and premature defoliation may occur and can result in 60–85% defoliation in susceptible cultivars [9,16], thus reducing vigor and affecting yield and quality even for the next season [11,17,18]. On fruits, symptoms generally start in late spring or early summer. Spots of 1 to 3 mm appear on the lenticels, sometimes surrounded by a reddish halo, often resulting in light tissue penetration. These spots are particularly visible during harvest time and postharvest storage and make the product unmarketable for fresh consumption or downgraded for juicing, resulting in significant losses to the grower [10]. It may cause soft rot, particularly when the skin has already been damaged by other means, especially mechanical wounds [16], or cracks around the apple calyx [19]. Fruit spot can cause losses between 10 and 40% of production depending on the year, farm and variety, with the Gala and Golden Delicious varieties being the most harmed.

The saprophytic phase of *Alternaria* spp. occurs during winter when the fungus overwinters as mycelium mainly in leaf residues on the orchard floor and also in twigs or dormant buds. During spring, spore production increases on dormant leaves under suitable conditions related to the increase of temperature combined with rainfall [20,21]. Factors such as water and wind favor the spread of conidia spores into the tree canopy, where they can colonize the growing leaves and fruits, producing primary infections with lesions of varying severity depending on the intensity [3,22,23]. Primary infection takes place one month after petal fall [10]. The disease progresses rapidly in optimum temperatures ranging from 25 to 31 °C and 5.1 h of wetting and symptoms can start appearing two days after infection [9]. The freshly emerging shoots are infected from about 20 days after bloom. Disease incidence and secondary infection increases during the growing season, where peak temperatures are combined with high rainfall and relative humidity. Disease progress on leaves and fruit continues to increase until the end of summer or beginning of autumn when defoliation occurs.

Due to the first symptoms generally appearing in late spring and developing during the summer until harvest, a high number of fungicide applications must be made even close to harvest, increasing the possibility of finding residues in the fruit. The chemicals recommended for control of apple leaf blotch and fruit spot are pyraclostrobin + boscalid, captan, mancozeb, fludioxonil and fluopyram + febuconazole. Moreover, the effectiveness of fungicide applications is not enough to reduce disease severity in some cases, especially under high inoculum pressure. Eradication of primary sources of inocula has shown to be a successful management option for other diseases [24,25] and may be a good option for the control of Alternaria leaf blotch and fruit spot of apple. Application of urea on fallen leaves, mulching, removal of weeds, discarding fallen apple litter from orchards, application of lime sulphur, and manual removal of leaf residues could reduce the sources of inocula in orchards [20]. Spores also reside on twigs and buds during the winter season. Protective spray of copper-based fungicide is recommended prior to development of new leaves during the end of autumn or early spring. Selective pruning of the canopy also reduces inocula present in twigs and buds in orchards [20].

Although, the disease cycle of leaf blotch and fruit spot of apple is poorly understood, it seems that the main source of overwintering inocula are the fallen leaves on the orchard floor. Thus, in the case of *A. mali*, the number of conidia detected was higher and had higher germination in leaves rather than in buds, suggesting the importance of leaves as an important source of primary inocula [26]. While, in *Alternaria* complex related with leaf blotch and fruit spot, the cumulative spore production is higher in leaf residue rather than in twigs and canopy leaves [27]. For this reason, an environmentally friendly strategy should be focused on reducing primary inocula production to increase control efficiency and to replace at least in part the synthetic phytosanitaries. According to this, the aim of this work was to optimize the control of *Alternaria* infection through two inoculum-management strategies, the removal of winter fallen leaves to reduce the availability of inocula, and the treatment of leaves with the biological agent *T. asperellum* to inhibit or prevent inocula development on fallen leaves. Trials were performed during three consecutive years in commercial orchards to determine if the disease control achieved by the standard fungicide sprays strategy was improved by the use of additional sanitation measures. This new strategy could mean a step forward on the way to much more sustainable apple production with the final goal of production of higher overall quality with the lowest environmental impact.

## 2. Materials and Methods

### 2.1. Field Trials Design and Conditions

Four trials were carried out in two apple orchards located in Catalunya in northeastern Spain during three consecutive years, specifically during 2019, 2020, and 2021. Orchards were selected that were naturally infected by Alternaria leaf blotch and fruit spot with a history of remarkable damage. The first orchard was of the Golden Reinders variety located in Garrigàs (42.19452, 2.97272), with trees grafted onto M9 and a 3.75 × 1.2 m plantation frame. The second orchard was of the Brookfield Gala variety located in Sant Pere Pescador (42.16517, 3.09509), with trees grafted onto M9 NAKB and a 3.8 × 1.0 m plantation frame. Both orchards had a central axis training system, irrigation by a drip system and were without anti-hail nets. During the assays, fertilization, pruning, herbicide and phytosanitary treatments were conducted following standards of integrated production used in commercial apple orchards of the region. For easy identification throughout the paper, trials have been codified (Table 1).

Two primary inoculum-management methods, leaf aspiration (ASP) and application of *T. asperellum* on the ground (TRI) were tested in comparison with a control where no inoculum management was performed (CNT). ASP consisted of completely removing leaves from the ground from February to middle of March depending on the trial (Table 2). The fallen leaves were collected by raking and vacuuming with a tractor-driven vacuum machine (John Deree TC125 Turf Collection System). Collected leaves were burnt in order to inactivate the *Alternaria* inoculum. TRI consisted of a single application onto the ground surface with the T34 strain of *T. asperellum* (5 × 10^4^ cfu/g) provided by Dr. María Isabel Trillas Gay from Biocontrol Technologies S.L (Barcelona, Spain). The application was made in the tree rows in April at a dose of 0.5 g/L at an application volume of 600 L/ha, when the temperature and humidity conditions were adequate. Depending on the trial, different strategies were applied (Table 2).

Each strategy was arranged in a single plot of approximately 1000 m^2^, that was randomly selected in each orchard. The dominant wind direction was considered at the time of distributing strategy plots, to avoid interference of sources of inocula. Each plot consisted of three rows of 80 m long in trial 1 and 90 m long in the other three trials. The external rows were considered buffer zones while the center row was where the evaluations were performed. Four replicates of ten trees were randomly selected and labeled in the central row for disease and spore release assessment.

Disease control during the growing season was based on fungicide applications. Fungicides were applied in all the plots independently of the inoculum-management strategy. Applications were made to preventively cover rain periods from June to August. The type of fungicides used were different in function for the risk period. Thus, the first rain in early June was covered with boscalid 25.2% + piraclostrobin 12.8% at 80 mL/hl (Bellis, BASF Española S.L.U, Barcelona, Spain) while the rest of the rains were covered with mancozeb 75% at 0.3 kg/hl (Vondozeb GD, UPL Iberia, S.A, Barcelona, Spain). Applications between 800 and 1000 L/ha were performed with a 3000-litre commercial Multi-fan sprayer (Teyme, Lleida, Spain). The number of applications was different in every trial and was dependent of the number of rainfalls during the risk period, from June to August (Table 2).

### 2.2. Spore Release

In order to determine the effect of leaf aspiration on the overwintering inocula, seasonal spore release was determined in the aspiration and control plots. Two spore traps consisting of microscope slides (2.6 cm wide and 7.6 cm long) painted with silicone solution (Lanzoni s.r.l., Bologna, Italy) and joined with a clothespin to 1 m bamboo were placed in each replicate of each plot. Thus, a total of 8 spore traps were installed per plot. These spore traps were placed in the field with the slides 75 cm above the ground and facing the main wind direction of the area. Sampling was carried out continuously for 2 years from 1 January 2020 to 31 December 2021, with slides changing every week. *Alternaria* spores are easily recognizable due to their typical morphology and were counted by direct microscopic observation (Carl Zeiss, Jena, Germany), using the methodology proposed by the Spanish Aerobiology Network, based on the observation of two longitudinal sections of a microscopic slide at a magnification of 400 [28]. Each section corresponds to a total area of 380 mm^2^. Means of the number of spores per cm^2^ were calculated and plotted in relation to the number of days. In addition, the area under the spore release curve (AUSRC) was calculated by the method of trapezoid integration [29].

### 2.3. Disease Assessment

The presence of lesions was evaluated in each replicate on 10 leaves of 20 shoots and on 100 fruits chosen at random and distributed on both sides of the trees. The incidence was assessed as the number of infected leaves or fruits over the total. For severity evaluation, each leaf or fruit was assessed according to the following semi-quantitative categorical severity index (SI): 0—no symptoms observed; (1)—up to 10% of the affected leaf surface or 1 lesion per fruit; (2)—10–50% of the affected leaf surface or 2–3 lesions per fruit; (3)—more than 50% of the affected leaf surface or more than 3 lesions per fruit. Then the following formula was used: $S=\overset{i}{\underset{i\to 1}{\sum }}\left(\frac{SI_{i}}{n\times 3}\right)\times 100$ (1), where *S* is the severity (0–100); *SI* is the disease severity index for each leaf or fruit; *i* is the number of infected fruits or leaves, *n* is the number of leaves or fruits; and *3* is the maximum level of severity.

### 2.4. Weather Conditions

In order to relate climatic factors to spore release dynamics in the orchards, weather data including daily minimum and maximum temperatures, relative humidity, wetness and rainfall were obtained from an automatic weather station placed in the experimental orchards. Temperature and relative humidity were measured every 10 min and wetness and rainfall every 20 s. Mean temperature, relative humidity, duration of wetness, and total rainfall were recorded every hour.

### 2.5. Data Analysis

All data are presented as mean ± standard deviation (SD). To statistically analyze results, Student’s *t* test and one way analysis of variance (ANOVA) were applied and significant differences (*p* < 0.05) among the treatments were determined using a post-hoc Tukey HSD test when necessary. Analyses were performed using the statistical package JMP (v16, SAS Institute Inc., Cary, NC, USA).

## 3. Results

### 3.1. Effect of Treatments on Spore Release

**Figure 1:**
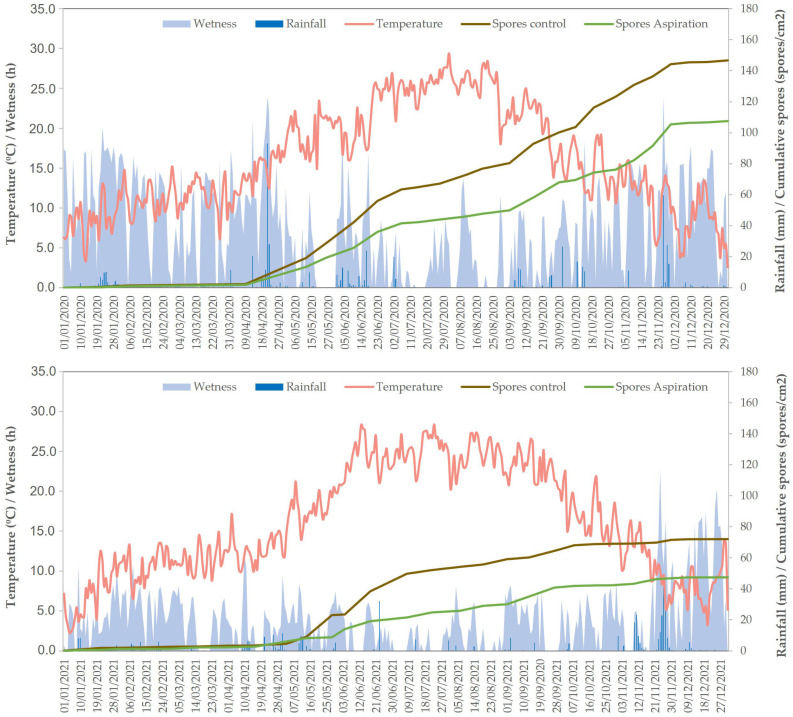
Dynamics of spore release and mean temperature, daily wetness duration and rainfall in trials 3 and 4 performed on 2020 and 2021, respectively.

Climatic conditions in the two years, where assessment of spore release was conducted, were very different taking into account the cumulative rainfall and the number of rainy days. The year 2020 was the rainiest, with a total of 639 mm of cumulative rainfall and 159 rainy days, while in 2021 the total cumulative rainfall was only 437.4 mm with 100 rainy days. The amount of rainfall and the rainy days affected the spore release; thus, the cumulative release of spores was higher during the year 2020 than in 2021 (Figure 1). Spore release started when the median temperature was above 12.5 °C and was associated with rain episodes. The period of maximum spore release, independent of the year, was between May and June, accumulating to 31.66% and 56.62% of the total of released spores in 2020 and 2021, respectively. During this period, the differences between released spores in the control plots and the aspiration plots were highest. After this period, the accumulation of spores slowed down during the months of July and August, with a slight increase in September, and finally stopped from October until December. In the case of the year 2020, an increase in spore release was observed due to the exceptional rainfall conditions between September and November. When the total amount of released spores was compared between control plots and aspiration plots on the basis of the AUSRC, significant reductions were observed in both years, with a similar reduction, 28.18% in 2020 and 29.07% in 2021 (Table 3). Thus, although the AUSRC was higher in 2020 because the spore release was greater throughout the season, the effect of spores release was the same in both years independent of the total amount of spores released.

**Figure 2:**
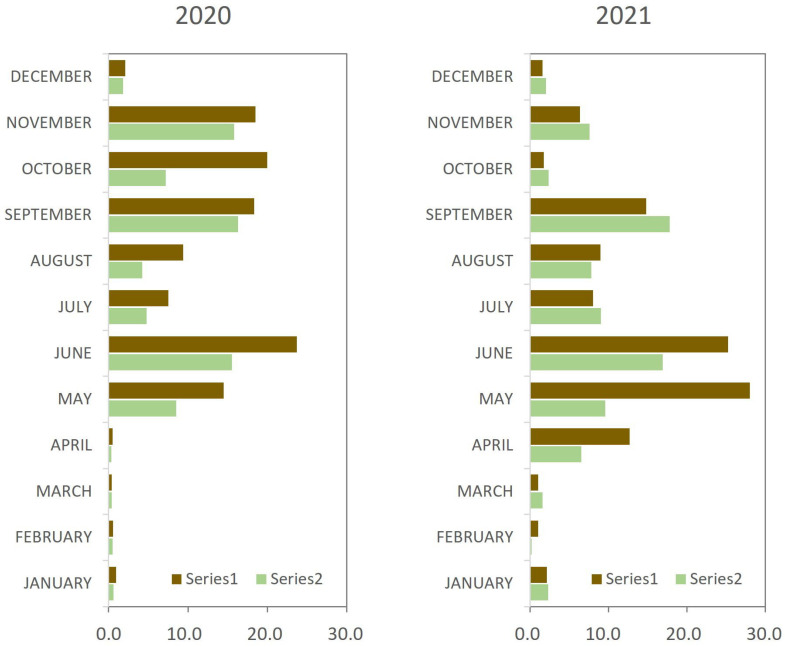
Spore release month by month in the control and in the aspiration plots in 2020 and 2021.

When the release of spores was analyzed month by month, differences between the control and aspiration plots were very clear in some month, such as May and June, where the number of spores released in the aspiration plot decreased by 39.22% and by 34.58% in 2020, and by 70.38% and by 59.29% in 2021, respectively. This reduction was very important and occurred during the period of maximum risk of infection. In addition, in most months, the release was lower in the aspiration plots, with some exceptions that coincide with periods of low spore release. Results also show low spore release in cold months, including January, February, March and December, where the amount of spores was residual (Figure 2).

### 3.2. Effects of Treatments on Alternaria Leaf Blotch and Fruit Spot

**Figure 3:**
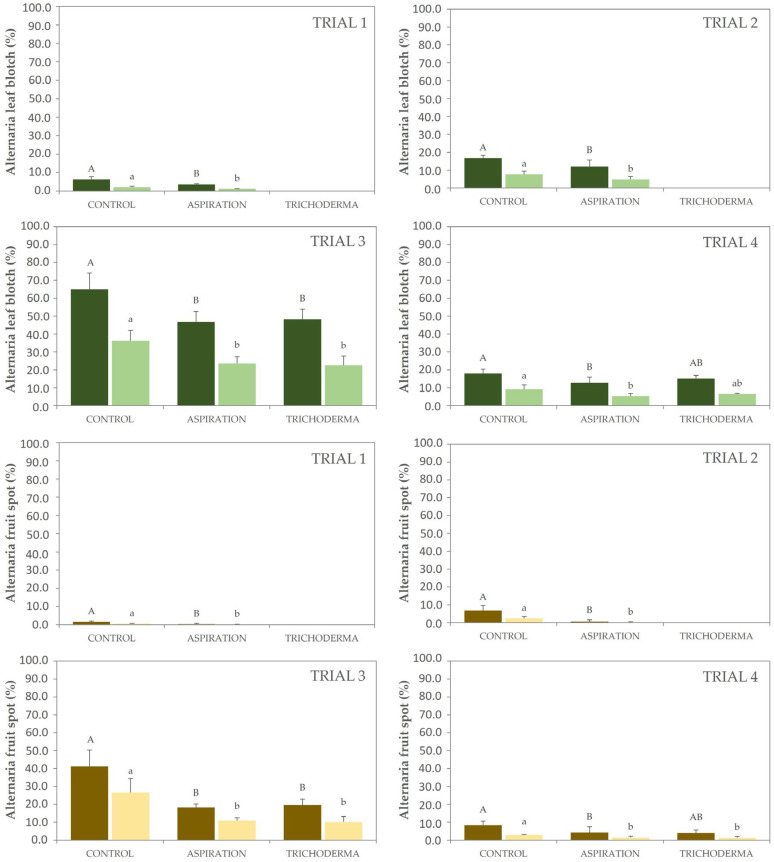
Incidence (dark plots) and severity (clear plots) of Alternaria leaf blotch (green series) and fruit spot (brown series) in commercial orchards where fallen leaves were collected by aspiration during winter or treated with *T. asperellum* during spring in comparison with controls without any inoculum management. Data correspond to the evaluations of 5 August 2019 for trial 1, 9 August 2019 for trial 2, 27 July 2020 for trial 3, and 29 July 2021 for trial 4. Data are presented as the mean of four replicates with the standard deviations (vertical bars). Different letters (capital letters for incidence and lowercase letters for severity) show significant differences between the treatments according to Student’s *t* test (*p* < 0.05) in trials 1 and 2, or Tukey’s test (*p* < 0.05, ANOVA, LSD) in trials 3 and 4.

The effects of different inoculum-management strategies on the development of Alternaria leaf blotch and fruit spot were tested in trials 1, 2, 3 and 4 (Figure 3). The level of natural infection in the different trials was different depending on the year and between varieties. In 2020, the incidence of Alternaria leaf blotch and fruit spot in golden varieties were too high with values around 65% and 40%, respectively. While in the rest of the years, 2019 and 2021, the incidence was moderate, with values in the control around 20% for leaf blotch and 8% for fruit spot. In contrast, the incidence in the Gala variety was too low, with incidences of 6% and 1% of leaf blotch and fruit spot, respectively. In all cases, severity was quite lower than incidence, indicating that infections were moderate, with few leaf surfaces affected and few spots per fruit. When the evolution of symptoms was analyzed in comparison with climatic conditions, the appearance of new spots was strongly related to episodes of rain (data not shown).

In trials 1 and 2, only aspiration of fallen leaves was tested in comparison with the control where no inoculum management was performed. In both assays, the leaf aspiration significantly reduced the incidence and severity of leaf blotch and fruit spot. The efficacy on reduction of fruit spot was higher with values above 80%, while the efficacy on the reduction of the leaf blotch was lower, with values around 40% in trial 1 and 30% in trial 2. In trials 3 and 4, both strategies were tested with good results, and a significant reduction of the incidence and severity of leaf blotch and fruit spot were observed in comparison with the control. Although, efficacies were lower than in trials 1 and 2. Again, the efficacies on the reduction of fruit spot were higher with values around 60% in trial 3 and 50% in trial 4. In contrast, the efficacies on the reduction of leaf blotch incidence and severity were slightly lower, with values around 30% in both assays. No differences were observed between the two inoculum-reduction strategies in any of the trials, showing in all cases similar efficacies.

## 4. Discussion

In recent years, Alternaria fruit spot has become one of the most important problems in apple production in the area of Girona in Spain. Its emergence has led to an increase of fungicide treatments and even so the production losses have been from 10 to 40% depending on the year, the orchard and the variety, with Gala and Golden being the most problematic. The disease has been expanding since 2009, arriving in 2017 to cause problems in around 20% of commercial apple orchards in Girona. Specifically, the explosion of this disease has been accompanied by an increase of between 20 and 30% in the number of treatments with fungicides. This increase is very significant, and furthermore these are concentrated very close to the harvest and have a direct effect on the residues on the fruit. This circumstance represents a serious obstacle to the aim of increasing the sustainability of exploitations, and obtaining fruit free of residue for commercial purposes. Moreover, in most years, the increase of fungicide applications has not represented a significant reduction in damage, in accordance with similar experience reported in Australia when the effectiveness of fungicide applications was often erratic in reducing disease severity and varied among regions [10]. Failure to control diseases may be due to high inoculum pressure in the orchards, but also due to the products used, which are probably not the most suitable, nor their positioning. Increasing knowledge of the identification of inoculum sources and also of the periods of maximum risk of infection is essential to attain satisfactory control of Alternaria fruit spot. According to spore release, results showed that release starts when temperatures are above 12.5 °C and ends when the temperatures drops below this value. Moreover, release is related to rain episodes indicating that rain splash helps in the release and projection of spores. In addition, when comparing released spores between 2020 and 2021, the difference was clear, being much higher in 2020 when the amount of rainfall, number of rainy days, and total wetness duration were higher. This evidence indicates that these climatic events can be considered important factors in increasing the incidence and severity during infection periods. These results are in agreement with findings for Alternaria brown spot of mandarin caused by *A. alternata* [30] and for *A. mali* in apple [17]. In this way, a comprehensive use of this information can be very useful to define risk episodes and the degree of severity, and therefore, the first step in the development of a forecasting model to predict infection episodes and help in the correct positioning of fungicides in a preventive strategy. Use of decision-support systems based on epidemic models have been demonstrated to be a very effective strategy to control fungal diseases, such as apple scab [31] and downy mildew and powdery mildew in grapevine [32], and it is a strategy to be explored and developed in the future.

When comparing the released spores in the control plots and in the aspiration plots, the amount of spores was lower in the aspiration plots, indicating that leaves are a reservoir of some disposable *Alternaria* spores, and probably one of the main inoculum sources as indicated by different authors [26,27]. Thus, the hypothesis is that *Alternaria* spores maturate in the leaf residue during autumn and winter, and probably early spring, generating the first spores that will be released during May and June. After this release, new spores are produced in infected leaves producing more spores that will be released during summer. This is supported by the fact that differences between spore release in the standard blocks and aspirated blocks are mainly in the period of May–June. After this period, light differences in spore release were observed between blocks. Historically this period of May–June has been related to the maximum risk of infection by *Alternaria*, thus limiting the release of spores during this period is important for a suitable control of infection. These results indicate that improving the management of the orchard to reduce the inocula of the pathogen through good management of leaves can help in the reduction of spore release and probably in disease control. This is in agreement with some reports that show good efficacy in the control of similar pathogens, like brown spot in pear caused by *Stemphylium vesicarium* (Wallr.) E.G. Simmons [33] or apple scab caused by *Venturia inaequalis* (Cooke) G. Winter in apple [34,35] using similar strategies focused primary on inoculum management. Therefore, it is necessary to use new control strategies based on reduction of the primary inocula to complement fungicide strategies. In this way, the findings from 3 year trials in commercial orchards showed that leaf litter management by aspiration and by *Trichoderma* application allowed a significant reduction in Alternaria leaf blotch and fruit spot development. The incidence and severity of the diseases in both leaves and fruits decreased significantly, with reductions between 50 and 80% of fruit spot and between 30 and 40% of leaf blotch depending on the year. These results support the idea that reducing the source of inocula by removing fallen leaves is a suitable strategy that can be used complementally to fungicides or biological control agent application in this area. Other studies have reported similar results in controlling other fungus diseases. Management of litter by leaf shredding and removal reduced the incidence and severity of apple scab in a French orchard by 50–80% and by about 90% in North Carolina [25,36], and successfully reduced apple scab ascospore production [34,37]. In pear it was reported that leaf shredding or removal were the most effective methods of reducing overwintering inocula of *Pleospora allii* (Rabenh.) Ces. & De Not and *S. vesicarium,* the causal agent of brown spot [33,38]. On the other hand, application of *Trichoderma* significantly decreased the incidence and severity of leaf blotch and fruit spot by around 50% and 20–30%, respectively. In agreement with this result, this biological control agent has shown the ability to reduce both overwintering inocula and conidia production of *S. vesicarium* and *P. allii* when it was applied to a pear orchard [39,40]. Moreover, *Trichoderma* inhibits the causal agent of apple ring rot *Botryosphaeria berengeriana* (De Notaris) and reduces the incidence on fruit [41], the causal agent of apple valsa canker *Valsa ceratosperma* (Tode) G.C. Adams & Rossman [42], and *Colletotrichum gloeosporioides* (Penz.) Penz. and Sacc, the cause of pre-harvest fruit drop in citrus [43]. In some of these studies, the mycoparasitic activity of *Trichoderma* was evidenced as a mechanism of action [41,42]. Although *Trichoderma* species are widely used in agriculture as biocontrol agents, their use to control *Alternaria* overwintering inocula in apples needs further studies due to its great potential. Considering that both tested strategies were applied in combination with phytosanitary treatments following the standards of integrated production used in commercial apple orchards of the region, it is suggested that the control of inocula source through alternative environmentally friendly strategies may act as an important complementary factor to fungicides, thus achieving the goal of reducing their use. This has been shown in previous studies performed in pear and apple orchards, in which also an alternative management was added to conventional fungicides [34,44]. Moreover, the consistent use of this strategy of inoculum management can be a good way to clean problematic orchards, gradually reducing the inoculum pressure. This can be an important aspect to take into account in organic orchards, where the limited number of fungicides make the efficient control of Alternaria fruit spot difficult.

## 5. Conclusions

The findings of this study point in the direction that sanitation of orchards through the elimination or the treatment with *Trichoderma* of the falling leaves reduces spore release mainly during the period between May and June. Moreover, this reduction is directly related to Alternaria leaf blotch and fruit rot reduction suggesting that this strategy based on the reduction of the primary inocula can be useful to complement a fungicide strategy.

## Figures and Tables

**Table 1:** Characteristics of trials performed for evaluating the effect of different sanitation treatments aimed at controlling Alternaria fruit spot of apple.

Trial	Year	Country	Orchard Location	Cultivar	Plot Size	Sanitation Strategy ^1^
1	2019	Spain	Sant Pere Pescador	Gala	2000 m^2^	ASP
2	2019	Spain	Garrigàs	Golden	2000 m^2^	ASP
3	2020	Spain	Garrigàs	Golden	3000 m^2^	ASP, TRI
4	2021	Spain	Garrigàs	Golden	3000 m^2^	ASP, TRI

^1^ ASP—leaf aspiration; TRI—application of *Trichoderma asperellum.*

**Table 2:** Treatments performed in orchard trials for evaluating the effect of different sanitation methods aimed at controlling Alternaria fruit spot of apple. Treatments included are no inoculum-management strategy (CNT) and aspiration strategy (ASP).

Trial	Year	Treatment	Action Date	Fungicide Application Dates ^1^	Disease Evaluations ^2^
1	2019	CNT	-	10/06; 12/07; 29/07	11/07; 09/08
		ASP	06-March		
2	2019	CNT	-	10/06; 12/07; 29/07; 21/08	11/07; 09/08
		ASP	06-March		
3	2020	CNT	-	09/06; 26/06; 08/07; 22/07; 09/08	27/07; 03/09
		ASP	04-Feb		
		TRI	14-April		
4	2021	CNT ASP TRI	- 23-March 03-May	18/06; 07/07; 20/07; 02/08	29/07; 13/09

^1^ Applications were done mainly with mancozeb 75% at 3.0 kg/ha to cover rain episodes. ^2^ Evaluations were performed according to the presence and the evolution of symptoms.

**Table 3:** Comparison of area under the spore release curve (AUSRC) obtained in the control plots and the aspiration plots in 2020 and 2021 by Student’s *t* test.

Trial	Year	AUSRC–CNT ^1^	AUSRC-ASP ^1^	F-Ratio	*p*-Value > F
3	2020	131.09	94.14	8.1021	0.0291
4	2021	63.40	44.97	6.2126	0.0470

^1^ CNT—control where no inoculum management was performed; ASP, leaf aspiration.

## Data Availability

Data is contained within the article.

## References

[1] Gao L.L., Zhang Q., Sun X.Y., Jiang L., Zhang R., Sun G.Y., Zha Y.L., Biggs A.R. (2013). Etiology of moldy core, core browning, and core rot of Fuji apple in China. Plant Dis..

[2] Johnson R.D., Johnson L., Kohmoto K., Otani H., Lane C.R., Kodama M. (2000). A Polymerase chain reaction-based method to specifically detect *Alternaria alternata* apple pathotype (*A. mali*), the causal agent of Alternaria blotch of apple. Phytopathology.

[3] Gur L., Reuveni M., Cohen Y. (2017). Occurrence and etiology of Alternaria leaf blotch and fruit spot of apple caused by *Alternaria alternata* f. sp. mali on cv. Pink Lady in Israel. Eur. J. Plant Pathol..

[4] Grove G.G., Eastwell K.C., Jones A.L., Sutton T.B., Feree D.C., Warrington I.J. (2003). Diseases of apple. Apples: Botany, Production and Uses.

[5] Roberts J.W. (1924). Morphological characters of *Alternaria mali*. J. Agric. Res..

[6] Wenneker M., Pham K.T.K., Woudenberg J.H.C., Thomma B.P.H.J. (2018). First report of *Alternaria arborescens* species complex causing leaf blotch and associated premature leaf drop of ‘Golden Delicious’ apple trees in the Netherlands. Plant Dis..

[7] Toome-Heller M., Baskarathevan J., Burnip G., Alexander B. (2018). First Report of apple leaf blotch caused by *Alternaria arborescens* complex in New Zealand. N. Z. J. Crop Hortic. Sci..

[8] Rotondo F., Collina M., Brunelli A., Pryor B.M. (2012). Comparison of *Alternaria* spp. collected in Italy from apple with *A. mali* and other AM-toxin producing strains. Phytopathology.

[9] Filajdic N., Sutton T.B. (1992). Influence of temperature and wetness duration on infection of apple leaves and virulence of different isolates of *Alternaria mali*. Phytopathology.

[10] Harteveld D.O.C., Akinsanmi O.A., Drenth A. (2013). Multiple *Alternaria* species groups are associated with leaf blotch and fruit spot diseases of apple in Australia. Plant Pathol..

[11] Fontaine K., Fourrier-Jeandel C., Armitage A.D., Boutigny A.L., Crépet M., Caffier V., Gnide D.C., Shiller J., Cam B.L., Giraud M. (2021). Identification and pathogenicity of *Alternaria* species associated with leaf blotch disease and premature defoliation in French apple orchards. PeerJ.

[12] Bulajic A. (1996). First report of *Alternaria mali* on apples in Yugoslavia. Plant Dis..

[13] Marschall K., Bertagnoll M. *Alternaria alternata*, causal agent of lenticel rot and leaf necrosis on apple in Italy [*Malus pumila* Mill.; South Tyrol]. Proceedings of the Atti delle Giornate Fitopatologiche.

[14] Vilardell P. (2018). Una nueva enfermedad causada por *Alternaria* afecta a plantaciones de manzana en Girona. Vida Rural..

[15] Generalitat de Catalunya de Departament d’Acció Climàtica, Alimentació i Agenda Rural *Estadístiques Defin*. *De Conreus*. https://agricultura.gencat.cat/ca/departament/estadistiques/agricultura/estadistiques-definitives-conreus.

[16] Jung K.-H. (2007). Growth inhibition effect of pyroligneous acid on pathogenic fungus, *Alternaria mali*, the agent of Alternaria blotch of apple. Biotechnol. Bioprocess Eng..

[17] Filajdic N., Sutton T.B., Walgenbach J.F., Unrath C.R. (1995). The influence of European red mites on intensity of Alternaria blotch of apple and fruit quality and yield. Plant Dis..

[18] Cooke T., Persley D., House S. (2019). Diseases of Fruit Crops in Australia.

[19] Stern R., Ben-Arie R., Ginzberg I. (2013). Reducing the incidence of calyx cracking in ‘Pink Lady’ apple using a combination of cytokinin 6-benzyladenine and gibberellins (GA4+7). J. Hortic. Sci. Biotechnol..

[20] Madhu G.S., Sajad U.N., Mir J.I., Raja W.H., Sheikh M.A., Sharma M.A., Singh D.B. (2020). Alternaria leaf and fruit spot in apple: Symptoms, cause and management. Eur. J. Biotechnol. Biosci..

[21] Rozo M.E.B., Pinto V.F., Pose G. (2019). Especies de *Alternaria* asociadas a cultivos de manzana y pera en la región del Alto Valle del Río Negro, Argentina. Cult. Cient..

[22] Linares C.D., Belmonte J., Canela M., de la Guardia C.D., Alba-Sanchez F., Sabariego S., Alonso-Pérez S. (2010). Dispersal patterns of *Alternaria* conidia in Spain. Agric. For. Meteorol..

[23] Harteveld D.O.C., Akinsanmi O.A., Chandra K., Drenth A. (2014). Timing of infection and development of *Alternaria* diseases in the canopy of apple trees. Plant Dis..

[24] Kim Y.C., Lee J.H., Bae Y.-S., Sohn B.-K., Park S.K. (2010). Development of effective environmentally-friendly approaches to control Alternaria blight and anthracnose diseases of Korean ginseng. Eur. J. Plant Pathol..

[25] Gomez C., Brun L., Chauffour D., Vallée D.D.L. (2007). Effect of leaf litter management on scab development in an organic apple orchard. Agric. Ecosyst. Environ..

[26] Filajdic N., Sutton T.B. (1995). Overwintering of *Alternaria mali*, the causal agent of Alternaria blotch of apple. Plant Dis..

[27] Harteveld D.O.C., Akinsanmi O.A., Dullahide S., Drenth A. (2014). Sources and seasonal dynamics of *Alternaria* inoculum associated with leaf blotch and fruit spot of apples. Crop Prot..

[28] Almaguer M., Díaz L., Fernández-González M., Valdéz E. (2020). Allergenic fungal spores and hyphal fragments in the aerosol of Havana, Cuba. Aerobiologia.

[29] Scherm H., Savelle A.T., Boozer R.T., Foshee W.G. (2008). Seasonal dynamics of conidial production potential of *Fusicladium carpophilum* on twig lesions in southeastern peach orchards. Plant Dis..

[30] Bassimba D.D.M., Mira J.L., Vicent A. (2014). Inoculum sources, infection periods, and effects of environmental factors on Alternaria brown spot of mandarin in mediterranean climate conditions. Plant Dis..

[31] Jamar L., Cavelier M., Lateur M. (2010). Primary scab control using a “during-infection” spray timing and the effect on fruit quality and yield in organic apple production. Biotechnol. Agron. Soc. Environ..

[32] Caffi T., Rossi V., Bugiani R. (2010). Evaluation of a warning system for controlling primary infections of grapevine downy mildew. Plant Dis..

[33] Llorente I., Vilardell A., Vilardell P., Pattori E., Bugiani R., Rossi V., Montesinos E. (2010). Control of brown spot of pear by reducing the overwintering inoculum through sanitation. Eur. J. Plant Pathol..

[34] Holb I.J. (2006). Effect of six sanitation treatments on leaf litter density, ascospore production of *Venturia inaequalis* and scab incidence in integrated and organic apple orchards. Eur. J. Plant Pathol..

[35] Beckerman J., Abbott C. (2019). Comparative Studies on the effect of adjuvants with urea to reduce the overwintering inoculum of *Venturia inaequalis*. Plant Dis..

[36] Sutton D.K., MacHardy W.E., Lord W.G. (2000). Effects of shredding or treating apple leaf litter with urea on ascospore dose of *Venturia inaequalis* and disease buildup. Plant Dis..

[37] Vincent C., Rancourt B., Carisse O. (2004). Apple leaf shredding as a non-chemical tool to manage apple scab and spotted tentiform leafminer. Agric. Ecosyst. Environ..

[38] Llorente I., Vilardell A., Montesinos E. (2006). Infection potential of *Pleospora allii* and evaluation of methods for reduction of the overwintering inoculum of brown spot of pear. Plant Dis..

[39] Moragrega C., Carmona A., Llorente I. (2021). Biocontrol of *Stemphylium vesicarium* and *Pleospora allii* on pear by *Bacillus subtilis* and *Trichoderma* spp.: Preventative and curative effects on inoculum production. Agronomy.

[40] Rossi V., Pattori E. (2009). Inoculum reduction of *Stemphylium vesicarium*, the causal agent of brown spot of pear, through application of *Trichoderma*-based products. Biol. Control..

[41] Kexiang G., Xiaoguang L., Yonghong L., Tianbo Z., Shuliang W. (2002). Potential of *Trichoderma harzianum* and *T. Atroviride* to Control *Botryosphaeria berengeriana* f. sp. *piricola*, the cause of apple ring rot. J. Phytopathol..

[42] Valetti L., Lima N.B., Cazón L.I., Crociara C., Ortega L., Pastor S. (2022). Mycoparasitic *Trichoderma* isolates as a biocontrol agent against *Valsa ceratosperma*, the causal agent of apple valsa canker. Eur. J. Plant Pathol..

[43] Vu T.X., Tran T.B., Hoang C.Q., Nguyen H.T., Do L.M., Dinh M.T., Thai D.H., Tran T.V. (2021). Potential of *Trichoderma* asperellum as a biocontrol agent against citrus diseases caused by *Penicillium digitatum* and *Colletotrichum gloeosporioides*. Int. J. Agric. Technol..

[44] Llorente I., Vilardell A., Vilardell P., Montesinos E. (2008). Evaluation of new methods in integrated control of brown spot of pear (*Stemphylium vesicarium*, teleomorph *Pleospora allii*). Acta Hortic..

